# OA17 An evolving case of Anti PM/Scl Anti body associated Aggressive Systemic Sclerosis

**DOI:** 10.1093/rap/rkac066.017

**Published:** 2022-09-28

**Authors:** Sunari Subasinghe, Sunethra Wijesooriya

**Affiliations:** Medway NHS Foundation Trust, Medway, United Kingdom; Medway NHS Foundation Trust, Medway, United Kingdom

## Abstract

**Introduction/Background:**

Scleroderma is an autoimmune systemic disease characterised by fibrosis of the skin and vasculopathy mainly affecting the digital arteries. However, it can have variable presentations overlapping with other connective tissue diseases (CTD). It is associated with distinct anti bodies which will determine the clinical phenotype and the prognosis of the disease. Therefore, it is important to ascertain the presence of these antibodies early in the disease. We present a case of limited cutaneous systemic sclerosis (LcSSc) associated with anti PM/Scl antibodies which evolved over time to the characteristic clinical phenotype denoted by the antibodies needing aggressive immunosuppressive treatment.

**Description/Method:**

A 60-year-old Caucasian lady was presented to vascular unit with a digital ulcer of the right ring finger following painful bluish discolouration. She had history of cervical cancer, bilateral cervical ribs and was a smoker. Vascular surgeons ruled out large/medium vessel disease and thoracic outlet obstruction. Raynaud syndrome (RS) was diagnosed and started on nifedipine.

3 months later she was admitted with progression of RS involving both hands and painful swelling of the fingers requiring iloprost infusions. Patient was referred to rheumatology team who detected synovitis of the hands and no other features of CTD. She was found to be positive for ANA 1:320-640 titre with a nucleolar pattern. DsDNA and ENA panel were negative. LcSSc was diagnosed.

Over a year she developed gradual onset skin thickening up to the metacarpophalangeal joints, weakness of the proximal muscles of the lower limbs (power 4/5) and symptomatic interstitial lung disease with HRCT findings in keeping with cellular nonspecific interstitial pneumonitis. She was found to be positive for PM/Scl 75 and PM/Scl100 antibodies. Her creatine kinase (CK) was elevated to 2259U/l and MRI confirmed myositis. Treatment decisions were made after liaising with a tertiary care centre.

She was started on prednisolone 30 mg daily with good initial response and mycophenolate was introduced while reducing steroids. However, she was unable to stop steroids completely as symptoms worsened requiring frequent dose escalations. Low dose methotrexate was added on but she was intolerant. In the meantime, her myositis and lung disease deteriorated further with involvement of the upper limbs, elevation of CK, new fibrotic changes in the HRCT and deteriorating lung function tests.  She was treated with Rituximab with good response in terms of both muscle power and respiratory symptoms. However, after a year, symptoms deteriorated and she is awaiting repeat rituximab.

**Discussion/Results:**

The presentation and the progression of the disease of our patient is important to understand especially how scleroderma can evolve over time and how patients’ antibody profiles can be used for early identification and management of various phenotypes.

Primary RS is unlikely to cause digital ulceration which was the initial presentation of our patient. She also had concomitant cervical ribs complicating the matter and the initial focus was on excluding proximal vascular insufficiency. She was referred to the rheumatology only at the point of worsening RS requiring iloprost infusions.

At diagnosis, she only had swollen fingers, synovitis and RS which was initially managed with vasodilators and low dose prednisolone. Over one and half years she developed myositis and ILD in keeping with NSIP pattern emphasizing the importance of close follow up of the patients with scleroderma as the major organ involvement occurs within the first 3 years of diagnosis.

Anti PM/Scl 75/100 is a myositis associated antibody that denotes an overlap syndrome characterised by LcSsc, myositis, synovitis, ILD and calcinosis, present in 5% of the patients with Scleroderma.  Our patient didn’t fulfil EULAR classification criteria for idiopathic inflammatory myositis probably due to the absence of muscle biopsy. Patients with anti PM/Scl 75/100 show a nucleolar pattern on ANA staining as in our patient and usually show better prognosis in terms of ILD. Nevertheless, our patient didn’t respond well to first line immunosuppressive medication (steroids and mycophenolate) and needed Rituximab.

The first presentation of myositis and ILD were treated with high dose prednisolone (30mg) which was a challenging decision due to high risk of scleroderma renal crisis associated with steroid treatment and it being common within the first 5 years of the diagnosis. Her blood pressure was closely monitored during the treatment.

**Key learning points/Conclusion:**

It is important to identify patients with RP secondary to underlying connective tissue disease and refer them early to the rheumatology services for further workup.

Close and regular follow up of patients with a new diagnosis of scleroderma within the first 5 years is of paramount importance as they are likely to develop new organ involvement as evidenced by above case.

Screening of patients with scleroderma for disease related lung involvement at the time of diagnosis with lung functions tests and HRCT irrespective of the respiratory symptoms will lead to early detection of the disease and prompt management.

Importance of autoimmune antibody profile especially scleroderma related anti bodies in identifying the clinical phenotype for early treatment and prediction of prognosis.

The importance of liaising with a tertiary care centre in managing complicated patients with connective tissue diseases and this is the only pathway which enables access to Rituximab via NHS England.

Being vigilant of scleroderma renal crisis with diligent blood pressure monitoring when steroids are used especially in higher doses (>15mg of prednisolone a day).

It is necessary to have further studies and come to a consensus among the rheumatology community on when to perform scleroderma related antibodies in a patient. It is unclear what percentage of these patients are positive for these antibodies at the outset of the disease which can be several years before the onset of the distinct symptoms of the clinical phenotype.

